# Correction: Inhibition of c-Jun N-Terminal kinase attenuates low shear stress–induced atherogenesis in apolipoprotein E–deficient mice

**DOI:** 10.1186/s10020-022-00539-9

**Published:** 2022-09-07

**Authors:** Juan Wang, Feng Shuang An, Wei Zhang, Lei Gong, Shu Jian Wei, Wei Dong Qin, Xu Ping Wang, Yu Xia Zhao, Yun Zhang, Cheng Zhang, Ming-Xiang Zhang

**Affiliations:** 1 Key Laboratory of Cardiovascular Remodeling and Function Research, Chinese Ministry of Education and Chinese Ministry of Public Health, Qilu Hospital of Shandong University, Jinan, Shandong China; 2 Department of Endocrinology, Qilu Hospital of Shandong University, Jinan, Shandong China

## Correction: Mol Med (2011) 17:990–999 https://doi.org/10.2119/molmed.2011.00073

Following publication of the original article (Wang et al. 2011), the authors found one error in Fig. 2C. The western blot image of β-actin in Fig. 2C was wrongly placed, which resulted in an overlap with the western blot image of β-actin in Fig. 7C. We have found the original western blot image of Fig. 2C in the saved raw data from three independent experiments. The representative western blot image of Fig. 2C showed relative VCAM-1 protein expression in different carotid artery regions (undist and low) in the mice models of NS and JNK-I groups (n = 6, each group). Please find below a corrected version of Fig. 2C.

**Fig. 2:**
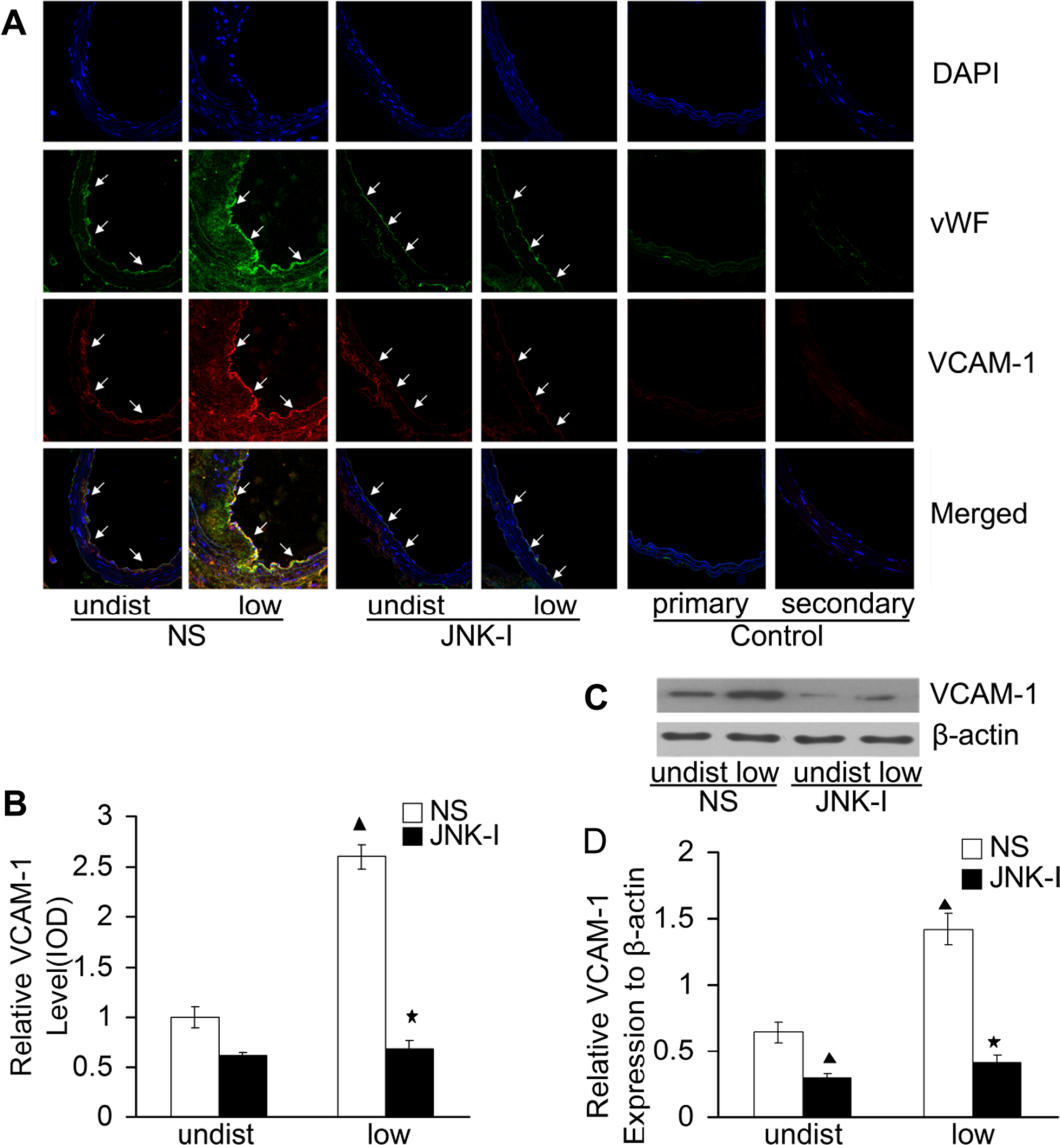
Relative VCAM-1 protein expression in different carotid artery regions. **A** Immuno-fluorescence analyses of VCAM-1 expression within vascular endothelium including primary and secondary antibody controls. Representative images acquired by laser scanning confocal microscopy (×400) showed vWF staining (green) of intact endothelium, VCAM-1 staining (red) and DAPI staining (blue) for cell nuclei. Bar = 100 μm; arrows denote the ECs. **B** Relative quantification of VCAM-1 expression in low and undisturbed (undist) regions by measuring the integrated optical density (IOD) of positive red area normalized to undisturbed flow region of the NS group. Western blot analysis of VCAM-1 in different regions (**C**) and quantification of protein expression of VCAM-1 relative to β-actin (**D**). Data are mean ± SD from six animals from low flow regions from experiments performed in triplicate. *P < 0.05 versus low regions in NS the group. ^▲^P < 0.05 versus undisturbed regions in the NS group
